# Effects of nutrition therapy on HbA1c and cardiovascular disease risk factors in overweight and obese patients with type 2 diabetes

**DOI:** 10.1186/s12937-018-0351-0

**Published:** 2018-04-07

**Authors:** Adham Mottalib, Veronica Salsberg, Barakatun-Nisak Mohd-Yusof, Wael Mohamed, Padraig Carolan, David M. Pober, Joanna Mitri, Osama Hamdy

**Affiliations:** 1 Joslin Diabetes Center, Harvard Medical School, One Joslin Place, Boston, MA 02215 USA; 2 Department of Nutrition and Dietetics, Faculty of Medicine and Health Sciences, Universiti Putra Malaysia, Selangor, Malaysia

**Keywords:** Clinical nutrition, Nutrition therapy, Lifestyle intervention, Diabetes management, Weight management

## Abstract

**Background:**

Nutrition Therapy (NT) is essential in type 2 diabetes (T2D) management. Standards of care recommend that each patient engages with a nutritionist (RDN) to develop an individualized eating plan. However, it is unclear if it is the most efficient method of NT. This study evaluates the effects of three different methods of NT on HbA1c and cardiovascular disease risk factors in overweight and obese patients with T2D.

**Methods:**

We randomized 108 overweight and obese patients with T2D (46 M/62F; age 60 ± 10 years; HbA1c 8.07 ± 1.05%; weight 101.4 ± 21.1 kg and BMI 35.2 ± 7.7 kg/m^2^) into three groups. Group A met with RDN to develop an individualized eating plan. Group B met with RDN and followed a structured meal plan. Group C did similar to group B and received weekly phone support by RDN.

**Results:**

After 16 weeks, all three groups had a significant reduction of their energy intake compared to baseline. HbA1c did not change from baseline in group A, but decreased significantly in groups B (− 0.66%, 95% CI -1.03 to − 0.30) and C (− 0.61%, 95% CI -1.0 to − 0.23) (*p* value for difference among groups over time < 0.001). Groups B and C also had significant reductions in body weight, body fat percentage and waist circumference.

**Conclusion:**

Structured NT alone improves glycemia in comparison to individualized eating plans in overweight and obese patients with T2D. It also reduces other important cardiovascular disease risk factors like body fat percentage and waist circumference.

**Trial registration:**

The trial was retrospectively registered at clinicaltrials.gov(NCT02520050).

## Background

Obesity and type 2 diabetes (T2D) are chronic diseases that reached pandemic proportions [1, 2]. Cardiovascular disease risk in patients with both T2D and obesity is significantly high and leads to increased morbidity and mortality [3, 4]. Physicians and professional societies agree that lifestyle changes that include nutrition therapy (NT) are the first-line therapy for patients with T2D [5–7]. The effectiveness of NT in reducing glycosylated hemoglobin (HbA1c) and cardiovascular disease risk factors in patients with diabetes was previously demonstrated in many studies and meta-analyses [8, 9]. Standards of care in diabetes recommend that each patient engages with a registered dietitian nutritionist (RDN) to develop an individualized eating plan [5, 6, 10]. In practice, patients and their healthcare providers became fully responsible in reaching an agreement on the best dietary caloric level and macronutrient distribution that fit patients’ needs [5, 6, 10]. This left many patients and their healthcare providers uncertain on how to best implement NT.

The aim of this study is to identify the optimal model of NT by evaluating the effects of three different methods of NT on HbA1c and other cardiovascular disease risk factors in overweight and obese patients with T2D.

## Methods

### Study population

We included female and male patients between 18–80 years of age who were diagnosed with T2D and were not treated with insulin but managed by stable doses of other diabetes medications for ≥3 months prior to enrollment, with HbA1c ≥7% and body mass index (BMI) ≥25 kg/m^2^. Patients on antihypertensive and/or cholesterol lowering medications were also managed by stable doses of these medications for ≥3 months prior to enrollment. We excluded pregnant women and patients with history of bariatric surgery, gastroparesis or patients who were actively enrolled in weight management programs. Study participants were recruited through advertisement in local media and clinic referrals. After screening, eligible participants signed the study consent form, which was approved by the Institutional Review Board.

### Study design

This is a prospective, randomized, three-arm study of 16 weeks duration. The study was conducted between April 2015 and June 2016. Eligible participants were randomized to three different methods of NT. Participants in group A followed the current standard of care recommendations [5, 6, 10] where they met with the study RDN to develop an individualized eating plan with objectives of lowering HbA1c and reducing weight through reduction of energy intake. Participants in this group received educational materials demonstrating the “Plate Method” and healthy eating [11]. This method teaches individuals to plan their meals such that one-third to one-half of their plate is filled with non-starchy vegetables, and the remainder of the plate is divided evenly between lean protein and starchy foods with an emphasis on healthy carbohydrate foods such as whole grains and starchy vegetables. Participants also received educational materials to guide them on making healthier choices within each food group. While there were no pre-specified targets for daily energy or macronutrient intake, dietary counseling for this group aimed at developing 2–5 individualized nutrition goals in order to improve HbA1c and reduce body weight. These goals were set while considering each participant’s motivation level, food preferences, current eating patterns, and ability to follow recommended dietary modifications. The study RDN provided support and follow-up on participants’ progress towards goals throughout the intervention at the scheduled study visits and follow-up phone calls.

Participants in groups B and C followed a well-defined, structured dietary plan according to the Joslin Nutrition Guidelines for overweight and obese patients with type 2 diabetes [12]. Participants were instructed to follow a hypocaloric dietary plan (1500 kcal/day for women, 1800 kcal/day for men) that included use of a commercially available diabetes specific nutrition formula (DSNF) 1–3 times per day within their caloric limit. The DSNF had 220 kcal/serving and contained 32.7% calories from fat, 40% calories from carbohydrate, and 27.3% calories from protein. The meal replacement was provided to participants free of charge. The meal plans provided approximately 40–45% calories from low-glycemic index carbohydrate, 1–1.5 g/kg of body weight from protein, and the rest of daily calories from fat with < 10% saturated fat. Sodium was limited to < 2300 mg sodium and fiber intake was adjusted to provide 14 g/1000 calories. All participants in groups B and C were provided with a dinner menu book containing 17 different recipes according the above macronutrients composition with detailed ingredients, nutrition facts and cooking instructions. Snack lists were also provided for one additional 150–200 calorie snack per day.

To investigate whether increased frequency of patient-RDN interaction affects study outcomes, group C received once weekly phone coaching and support provided by an RDN. The purpose of these calls was to motivate them to adhere to the nutritional intervention, provide guidance on implementing the structured dietary plan, and answer questions that may arise during intervention.

Study participants were asked to maintain their baseline activity level without any change throughout the study period and were not given any specific exercise or behavioral recommendations and were not asked to keep an exercise log in order to narrow the study intervention variables to that related to NT alone. All participants came to the clinical research center for three study visits (baseline, 8 weeks and 16 weeks) during which they spent one hour with the study RDN. All participants received two 15-min follow-up phone calls (at weeks 4 and 12) from the study RDN to provide dietary advice, promote adherence and address any questions or concerns. Before the first baseline visit, all participants were asked to complete a 3-day food log; recording all foods and beverages consumed on any 2 random week-days and 1 weekend day during the week prior to the visit. Participants in group A were asked to complete a 3-day food log during the week prior to their second and final study visits. Participants in groups B and C were asked to record their daily food and frequency of DSNF intake in their dietary log books throughout the 16 weeks of intervention.

Participants were instructed to record amounts of foods consumed using household measurements (measuring cups, measuring spoons, etc.) and to record weights or volumes of food or beverages for packaged, individual-serving foods. If participants consumed foods outside the home, they were instructed to estimate portion. The food logs had specified sections in which to record a description of the food or beverage consumed, how the food was prepared, and if there was any oils/fats, salt, sugar, or other condiments or sauces added to the food or beverage either during preparation or just prior to consumption. Participants were prompted by instructions on the food log to record the time and occasion of each meal, snack, or beverage. The research dietitian reviewed all food records with each participant to clarify portion sizes and preparation method, and probe for additional food and beverages consumed at and between each recorded eating occasion, and otherwise clarify errors, unclear descriptions, and questionable entries [13].

### Study procedures

Anthropometric measurements and blood samples were taken at each visit after an overnight fast. Blood pressure was measured in the seated position. Body weight was measured using a calibrated scale (Tanita BWB–800, Japan). Measurement of body composition was done using professional version of bioelectrical impedance analyzer (Tanita TBF–215, Japan). Visceral fat was measured using a validated bioelectrical impedance device (Tanita, Viscan AB–140, Japan) and was expressed in arbitrary units ranging from 1 to 59. Height was measured without shoes. Waist circumference was measured just above the hip bone and hip circumference was measured around the maximum circumference of the buttocks. Dietary macronutrients composition was assessed by analyzing food logs from the three study visits using the Food Processor Diet & Nutrition Analysis Software (version 10.15.41, 2015, ESHA Research, Salem, OR, USA). These analyses include total energy intake, average macronutrient values and percent energy intake from carbohydrate, total fat, saturated fat, protein and total dietary fiber per day. Insulin sensitivity was calculated using the homeostatic model assessment (HOMA-IR) equation from fasting plasma glucose and serum insulin at baseline and after 16 weeks [14].

### Outcome measures

The primary outcome for this study was the effect of NT on HbA1c after 16 weeks of intervention. Secondary outcomes include the change in body weight, body composition, visceral fat, waist and hip circumference, blood pressure, lipid profile, fasting glucose, insulin, c-peptide, insulin sensitivity (HOMA-IR), urinary microalbumin/creatinine ratio, high-sensitivity C-reactive protein (hs-CRP) and dietary macronutrients values.

### Randomization and masking

Randomization was conducted using a computer generated sequence with block design to ensure even distribution of study participants among intervention groups. Study investigators, RDN, participants and staff conducting assessments were not masked to treatment assignment. However, the study statistician was masked to treatment assignment to minimize bias during data analysis.

### Statistical analysis

All statistical analyses were conducted using SAS version 9.4 for Windows (SAS Institute, Cary, NC, USA 2012). Statistical significance was set a priori at *p* < 0.05. Primary analysis was of the as-randomized/intention-to-treat population. Demographic and baseline subject characteristics were assessed by general linear model (analysis of variance (ANOVA); PROC GLM). Change in outcomes over time was assessed by linear mixed effects model (analogous to repeated-measures ANOVA; PROC MIXED) which provides a flexible, likelihood-based approach to treating missing data and within-subject correlation in longitudinal studies.

In secondary analyses, we evaluated the effects of possible covariates (e.g. BMI) on study outcomes and found no significant effects of any candidate covariate nor any interaction with the main effects of interest. Addition of covariates to the model did not change the *p*-values of the regression coefficients for the main effect and did not reverse their sign, so we present the results of unadjusted analyses. The study was powered for a drop-out rate of 20%, which is common for nutrition intervention studies.

## Results

### Participant flow and baseline characteristics

**Fig. 1:**
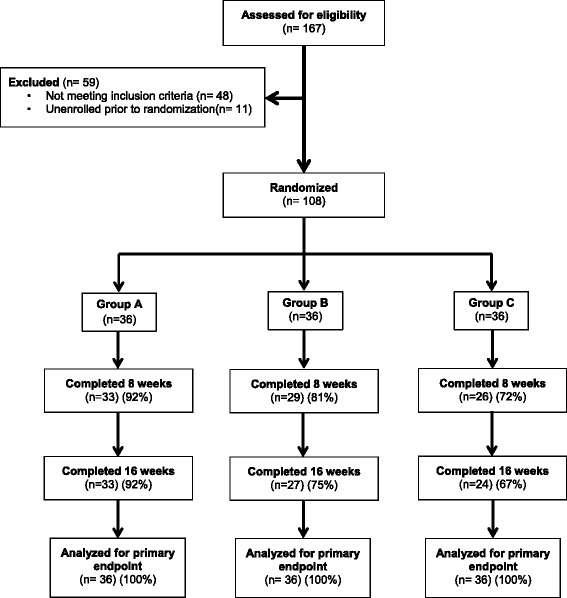
Flow diagram of study enrollment

Between May 2015 and January 2016, 167 individuals were screened for eligibility. Of which, 108 participants were randomized to the three study groups (Fig. 1). The study included 46 males and 62 females with mean (±SD) age of 60 ± 10 years. At baseline, study participants had mean HbA1c of 8.07 ± 1.05% and had mean diabetes duration of 11 ± 7 years; mean initial body weight of 101.4 ± 21.1 kg, mean BMI of 35.2 ± 7.7 kg/m^2^, and 24 (22.2%) subjects had a BMI of < 30 kg/m^2^. At baseline, there were no significant differences in any of these parameters among the three groups. Detailed baseline characters are shown in Table 1. Eighty-four participants completed the 16 weeks of follow-up (22% attrition rate); however, data from all 108 participants were included in our analysis. A per-protocol analysis of those subjects who completed all study requirements, and assessments of simple imputation of missing data (e.g. last observation carried forward) found no effect on the direction or significance of any conclusions, so we present the results for the planned intention-to-treat analysis. We found this to be appropriate since most of the drop-outs occurred before the second (week 8) study visit (Fig. 1). The drop-out rate was relatively higher in groups B and C compared to group A (*p* < 0.05 for difference among groups). Main causes of drop-out included time constraints, inability to follow the dietary plan and development of gastrointestinal symptoms thought to be related to DSNF consumption.

**Table 1:** Demographics and baseline characteristics of the study participants

	Group A (*n* = 36)	Group B (*n* = 36)	Group C (*n* = 36)	*P* value
Age (years)	57 (10)	61 (10)	61 (9)	0.14
Sex (male)	36%	44%	47%	0.62
Diabetes duration (years)	11 (10)	11 (6)	11 (6)	0.98
Number of diabetes medications	1.6 (0.8)	1.9 (0.9)	2.0 (1.1)	0.16
Race				
Asian	0%	3%	6%	0.77
Black	47%	19%	19%	< 0.05
Hispanic	6%	3%	8%	0.87
Non-Hispanic white	47%	69%	58%	0.18
Other/Unreported	0%	6%	8%	0.37
HbA1c (%)	8.15 (1.02)	8.17 (1.21)	7.99 (0.91)	0.44
Fasting plasma glucose (mmol/L)	10.1 (3.9)	9.8 (3.2)	8.7 (1.9)	0.14
Fasting insulin (pmol/L)	133 (88)	151 (108)	150 (110)	0.69
HOMA-IR	9.5 (10.1)	9.4 (7.2)	8.9 (8.6)	0.95
Total cholesterol (mmol/L)	5.09 (1.10)	4.45 (1.27)	4.08 (1.21)	0.38
LDL cholesterol (mmol/L)	2.43 (0.95)	2.47 (1.06)	1.95 (0.67)	< 0.05
HDL cholesterol (mmol/L)	1.18 (0.28)	1.13 (0.23)	1.20 (0.33)	0.51
Triglycerides (mmol/L)	1.72 (0.76)	1.89 (1.10)	1.79 (1.00)	0.77
Albumin/creatinine ratio (mg/mmol)	2.89 (3.54)	2.66 (3.87)	3.97 (10.58)	0.69
hsCRP (nmol/L)	53.8 (91.9)	41.0 (63.3)	37.2 (34.7)	0.55
Body weight (kg)	101.2 (20.7)	105.4 (25.3)	97.5 (16.3)	0.29
BMI (kg/m^2^)	35.4 (7.1)	36.4 (9.4)	33.9 (6.1)	0.36
Body fat (%)	41.2 (10.0)	42.5 (8.5)	39.9 (9.3)	0.52
Visceral fat (arbitrary units)	16.2 (5.8)	18.2 (7.1)	16.9 (5.0)	0.44
Waist circumference (cm)	117.1 (13.2)	121.9 (17.3)	117.1 (12.3)	0.11
Hip circumference (cm)	120.7 (15.9)	124.8 (20.3)	118.2 (11.2)	0.18
Wait/hip ratio	0.97 (0.07)	0.98 (0.07)	0.99 (0.08)	0.82
Systolic blood pressure (mmHg)	134 (15)	131 (18)	132 (14)	0.65
Diastolic blood pressure (mmHg)	72 (10)	72 (8)	71 (9)	0.8
Daily caloric intake (kcal)	1944 (699)	1943 (623)	1993 (813)	0.95
Carbohydrate intake (g)	216.9 (94.7)	206.6 (81.7)	223.3 (89.0)	0.73
Total fat intake (g)	85.1 (37.8)	88.2 (32.9)	82.8 (39.8)	0.83
Saturated fat intake (g)	27.7 (12.3)	28.0 (14.6)	27.4 (16.0)	0.98
Protein intake (g)	82.6 (22.4)	84.3 (24.9)	90.9 (32.5)	0.4
Fiber intake (g)	16.4 (5.4)	19.6 (6.0)	19.0 (6.6)	0.07

Data are mean (SD) or percentage. *p* values from Fisher’s Exact Test among groups at baseline. Group A: individualized nutrition therapy, Group B: Structured nutrition therapy, Group C: Structured nutrition therapy + weekly phone support

### Laboratory changes

**Fig. 2:**
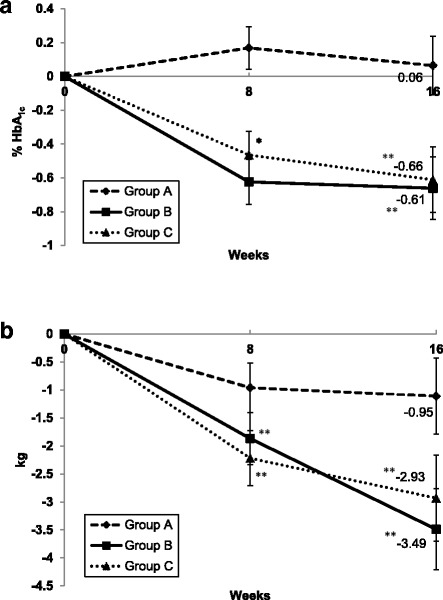
Change in HbA1c (**a**) and body weight (**b**) from baseline in response to different methods of nutrition therapy. Values are mean ± SEM. Group A: individualized nutrition therapy, Group B: Structured nutrition therapy, Group C: Structured nutrition therapy + weekly phone support. *n* = 36 in each group. * *p* < 0.01 and ** *p* < 0.001 compared to baseline

After 16 weeks, % HbA1c did not change from baseline in group A, but decreased significantly in groups B (− 0.66%, 95% CI -1.03 to − 0.30) and C (− 0.61%, 95% CI -1.0 to − 0.23) (*p* < 0.001 for difference among groups) (Table 2) (Fig. 2a). This difference among groups remained significant (*p* < 0.001) after controlling for age, baseline body weight, duration of diabetes, and change in body weight.

**Table 2:** Change after 16 weeks in metabolic, anthropometric and dietary intake parameters in response to three different methods of nutrition therapy

	Group A (*n* = 36)	Group B (*n* = 36)	Group C (*n* = 36)	*P* value^†^
HbA1c (%)	0.06 (−0.28 to 0.41)	− 0.66 (− 1.03 to − 0.30)^***^	−0.61 (− 1.0 to − 0.23)^**^	< 0.001
Fasting plasma glucose (mmol/L)	− 0.5 (− 1.5 to 0.6)	− 0.9 (− 2.0 to 0.2)	−0.7 (− 1.8 to 0.4)	0.09
Fasting insulin (pmol/L)	− 12 (− 37 to 12)	− 18 (− 6 to 1)	− 5 (−9 to − 1)^*^	0.76
HOMA-IR	− 2.1 (− 4.4 to 0.3)	− 1.9 (− 4.3 to 0.5)	− 2.8 (− 5.2 to − 0.3)^*^	0.51
Total cholesterol (mmol/L)	−0.07 (− 0.29 to 0.16)	0.06 (− 0.18 to 0.30)	0.11 (− 0.14 to 0.37)	0.50
HDL-cholesterol (mmol/L)	0.02 (− 0.03 to 0.07)	0.02 (− 0.04 to 0.08)	0.07 (0.01 to 0.13)^*^	0.28
LDL-cholesterol (mmol/L)	−0.06 (− 0.25 to 0.13)	0.05 (− 0.16 to 0.26)	0.10 (− 0.12 to 0.33)	0.71
Triglycerides (mmol/L)	− 0.05 (− 0.27 to 0.17)	−0.18 (− 0.41 to 0.06)	−0.09 (− 0.33 to 0.16)	0.31
Albumin/creatinine ratio (mg/mmol)	0.50 (− 1.92 to 2.92)	− 0.23 (− 2.72 to 2.27)	− 0.51 (− 3.08 to 2.07)	0.63
hsCRP (nmol/L)	9.2 (− 30.0 to 48.3)	14.4 (− 27.8 to 56.5)	− 6.2 (− 50.6 to 38.9)	0.81
Body weight (kg)	−1.11 (− 2.46 to 0.23)	− 3.49 (− 4.93 to − 2.05)^***^	− 2.93 (− 4.45 to − 1.42)^***^	0.11
BMI (kg/m^2^)	−0.43 (− 0.92 to 0.06)	−1.26 (− 1.78 to − 0.74)^***^	−1.06 (− 1.61 to − 0.52)^***^	0.13
Body fat (%)	−0.1 (− 0.9 to 0.6)	−1.6 (− 2.4 to − 0.8)^***^	−1.2 (− 2.0 to − 0.3)^**^	< 0.05
Visceral fat (arbitrary units)	0.01 (− 1.0 to 0.9)	−1.2 (− 2.2 to − 0.2)^*^	−0.3 (− 1.3 to 0.8)	0.40
Waist circumference (cm)	−0.4 (−2.3 to 1.5)	− 5.0 (− 7.0 to − 2.9)^***^	−2.9 (− 5.0 to − 0.8)^**^	< 0.01
Hip circumference (cm)	−1.8 (− 3.6 to − 0.1)^*^	−3.6 (− 5.2 to − 1.7)^***^	− 2.9 (− 4.8 to − 1.0)^**^	0.37
Wait/Hip ratio	0.01 (−0.01 to 0.03)	− 0.01 (− 0.03 to 0.01)	0.00 (− 0.02 to 0.02)	0.14
Systolic blood pressure (mmHg)	3 (− 2 to 8)	7 (2 to 13)^***^	0 (−6 to 6)	< 0.05
Diastolic blood pressure (mmHg)	3 (0 to 7)	1 (− 3 to 5)	3 (−2 to7)	0.41
Daily energy intake (kcal)	− 206 (− 394 to − 18)^*^	− 269 (− 469 to − 69)^**^	− 382 (− 586 to − 177)^***^	0.40
Carbohydrate intake (g)	−33.4 (− 60.4 to − 6.3)^*^	−29.7 (− 58.6 to − 0.9)^*^	−50.3 (− 79.7 to − 20.9)^**^	0.62
% Energy from carbohydrates	−1.4 (− 4.2 to 1.4)	− 0.5 (− 3.5 to 2.6)	−2.9 (− 6.0 to 0.3)	0.50
Total fat intake (g)	− 9.9 (− 19.8 to − 0.01)^*^	− 18.6 (− 29.2 to − 8.0)^***^	−16.9 (− 27.7 to − 6.1)^**^	0.16
% Energy from total fat	−0.2 (− 3.0 to 2.5)	−4.2 (− 7.2 to − 1.2)^**^	−1.2 (− 4.3 to 1.9)	0.10
Saturated fat intake (g)	−4.3 (− 7.7 to − 0.9)^*^	−10.1 (− 13.7 to − 6.4)^***^	−9.3 (− 13.1 to − 5.6)^***^	< 0.05
% Energy from saturated fat	−0.9 (− 2.1 to 0.3)	−3.3 (− 4.6 to − 2.1)^***^	−2.7 (− 4.0 to − 1.3)^***^	< 0.01
Protein intake (g)	1.3 (− 6.9 to 9.5)	11.0 (2.1 to 19.9)^*^	0.4 (− 8.8 to 9.7)	0.29
% Energy from protein	1.6 (− 0.3 to 3.5)	4.8 (2.8 to 6.8)^***^	4.2 (2.1 to 6.2)^***^	< 0.001
Fiber intake (g)	1.6 (− 0.5 to 3.8)	4.0 (1.6 to 6.4)^**^	2.7 (0.2 to 5.2)^*^	0.58

Values are mean (95% CI). *hsCRP* high-sensitivity C-reactive protein. ^*^p < 0.05, ^**^p < 0.01, ^***^p < 0.001 compared to baseline. ^†^*p* value for group*time effect. Group A: individualized nutrition therapy, Group B: Structured nutrition therapy, Group C: Structured nutrition therapy + weekly phone support

HDL-cholesterol increased significantly in group C compared to baseline (*p* < 0.05); however, total serum cholesterol and LDL-cholesterol did not change in any of the three groups (Table 2). No change was seen in fasting plasma glucose in the three groups, but fasting serum insulin and insulin sensitivity (HOMA-IR) improved in group C but these changes were not significant among groups (Table 2). No change was also seen in hsCRP and urinary microalbumin/creatinine ratio among the three groups.

### Anthropometric changes

Body weight did not change from baseline in group A, but decreased significantly in groups B (− 3.49 kg, 95% CI -4.93 to − 2.05) and C (− 2.93 kg, 95% CI -4.45 to − 1.42) (Table 2) (Fig. 2b). The change in body weight among groups was not statistically significant. Similar changes were also seen in participants’ BMI (Table 2).

Percentage body fat did not change from baseline in group A, but decreased significantly in groups B (− 1.6%, 95% CI -2.4 to − 0.8) and C (− 1.2%, 95% CI -2.0 to − 0.3) (*p* < 0.05 for difference among groups). Waist circumference did not change from baseline in group A, but decreased significantly in groups B (− 5.0 cm, 95% CI -7.0 to − 2.9) and C (− 2.9 cm, 95% CI -5.0 to − 0.8.) Change in waist circumference among groups was significant (*p* < 0.01). Hip circumference decreased significantly in the three groups; however, change among groups was not significant (Table 2). Visceral fat levels decreased significantly in group B only (*p* < 0.05); however change among groups was not significant (Table 2).

There was a small, yet significant, increase in systolic blood pressure in group B but it did not change in groups A and C (Table 2). Diastolic pressure did not change in any of the three groups.

### Dietary changes

**Fig. 3:**
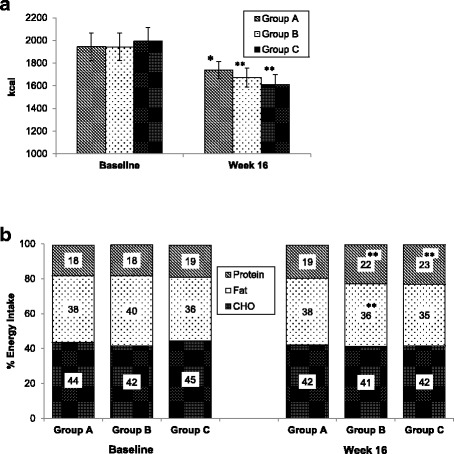
Change in: mean daily energy intake (**a**); percentage of energy intake from carbohydrates (CHO), fat and protein (**b**) in response to different methods of nutrition therapy. Group A: individualized nutrition therapy, Group B: Structured nutrition therapy, Group C: Structured nutrition therapy + weekly phone support. *n* = 36 in each group. * *p* < 0.05, ** *p* < 0.01, compared to baseline

Total energy intake was significantly lower in all groups compared to baseline with no difference among them (Table 2) (Fig. 3a). Similarly, all three groups significantly decreased their carbohydrate and total fat intake compared to baseline with no differences among groups (Table 2). All three groups significantly decreased their saturated fat intake compared to baseline; however, this reduction was higher in groups B and C (*p* < 0.05). Groups B and C significantly increased their fiber intake compared to baseline; however, this change was not significantly different among groups. Additionally, group B participants increased their protein intake compared to baseline; however, this change was not significantly different among groups.

Changes in the proportion of energy intake from carbohydrates and total fat were not different between groups. However, the proportion of energy intake from protein significantly increased in groups B (4.8%, 95% CI 2.8 to 6.8) and C 4.2 (2.1 to 6.2) compared to group A (*p* < 0.001) (Table 2) (Fig. 3b). Additionally, groups B and C significantly reduced their proportion of energy intake from saturated fat compared to group A (*p* < 0.01) (Table 2).

## Discussion

This is a head-to-head comparison between the recommended personalized NT approach and structured NT in patients with T2D. This study demonstrated that all three methods of NT effectively improved diet quality in overweight and obese patients with diabetes. All three interventions reduced daily energy intake, total fat, saturated fat and total carbohydrates compared to baseline. However, the structured dietary intervention was superior in helping participants significantly improve their glycemic control. An HbA1c reduction of 0.61–0.66%, as seen in the structured NT groups of this study, is similar to what was observed after few of the commonly used diabetes medications [15]. This reflects the prominent value of this NT method in managing T2D among this population. It is impressive to see these results in patients who had T2D for a long duration of an average (±SD) 11 ± 7 years and with baseline HbA1c of an average (±SD) 8.07 ± 1.05%. Moreover, structured NT significantly reduced body weight by 2.9–3.5 kg. It also reduced waist circumference and percentage body fat even at a similar daily energy intake level of individualized NT; without any modification in physical activity or medication change. It has been long perceived that lifestyle improves glycemic control and body weight [16, 17]. However, this notion was challenged by a recent meta-analysis of lifestyle intervention studies that showed collectively an average weight reduction of < 5%, which the authors considered a small magnitude that is not enough to improve metabolic parameters [18]. In contrast, this study demonstrates that it may be possible to significantly lower HbA1c and achieve modest weight reduction of around 3% through structured NT alone. Considering that diabetes medications were stable during the entire study and that participants were advised not to change their routine physical activity, it is more likely that observed metabolic improvements were attributed to structured NT.

Participants in the Look AHEAD trial were given daily energy intake goals of 1200–1500 or 1500–1800 calorie depending on their starting weight. As part of the dietary intervention, participants were instructed to use meal replacements 3 times daily, replacing breakfast and lunch with a meal replacement beverage and replacing one snack with a meal replacement bar. Participants could choose from four meal replacements that were provided free of charge [19]. However, later analysis identified a total of 38 different meal replacement beverages consumed by participants. The meal replacement beverages had an average of 180 calories each, and ranged in macronutrient contents as follows: 13–37.9% calories from fat, 24.9–70.6% calories from carbohydrates, and 17.3–50.1% calories from protein [20]. While instructed to use a DSNF 2–3 times/day, participants in our study used it 2 times/day on average. DSNFs were used to replace breakfast, lunch, and one snack if using a third one is used.

Although daily energy intake at baseline and at 16 weeks was not different among groups and calorie reduction was similar, structured NT resulted in better outcomes. Several explanations could be presumed. Participants in structured NT groups consumed more protein and fiber, which are known to improve postprandial blood glucose levels [21–24]. Use of structured menus and snacks provided consistent ratio of macronutrients and energy intake per meals that might reduce blood glucose variability. Use of DSNFs, within the total daily energy intake, had its own benefit on glycemic control, [25] insulin and GLP-1 secretion, [21] and body weight [26, 27]. Taken together, these factors might explain the improvement in glycemic control, body weight, body composition and insulin sensitivity in response to structured NT.

A recent systematic review of different dietary interventions concluded that there is not enough evidence to suggest a preferable diet for managing overweight and obese patients with T2D after controlling for weight loss [28]. Results of our study demonstrates that changes in glycemic control among groups was significant over time even after controlling for changes in body weight, baseline body weight, age and diabetes duration. This strongly suggests that observed improvement in glycemic control is, at least in part, due to the macronutrient composition provided within the structured NT plan.

While optimal frequency of RDN-patient communication is unknown, our findings suggest that weekly communication between study participants and RDN has no additional benefit over the current and typical follow up practice.

This study had some limitations. It was limited to overweight and obese patients, so it is unknown if similar results can be reproduced in lean patients with type 2 diabetes. It also did not include patients treated with insulin. However, frequent titration of insulin in any nutrition study may impact glycemic outcomes and adds a strong variable that may affect study results. Group A had 92% of its participants complete all 16 weeks of follow up, while groups B and C had a significantly lower completion rate. The higher attrition rate observed in groups B and C may be attributed to the highly structured nature of the intervention, including the use of DSNFs, which was not sustainable for some participants. Although utilization of 3-day food records was validated in multiple studies, this method has its limitations with regards to characterizing subjects’ usual diet due to within-person variations of day-to-day food intake. Based on this we suggest that patients first start with a structured NT plan when initiating NT. However, a more personalized eating plan might be more suitable for those who are not able to adhere to structured NT. The study was conducted at single diabetes center in an urban location, so it is unknown if different population or other location may have an impact on the study results. The follow up period was limited to 16 weeks, so it is unknown if these improvements will persist for longer duration which warrants a study with long-term follow up.

## Conclusion

In conclusion, an RDN-provided structured nutrition therapy which includes a macronutrient composition that is higher in protein and lower in saturated fat within a pre-specified level of energy intake, in addition to the use of menus, snacks lists, a diabetes-specific nutritional formula and keeping a daily food record results in the lowering of important cardiovascular disease risk factors, namely HbA1c, body fat percentage and waist circumference, compared to individualized meal plans offered to overweight and obese patients with type 2 diabetes. This study adds additional evidence to support the important role of RDNs in conducting NT using a structured plan. Additionally, weekly RDN phone support does not have additional benefit in patients following structured NT. Further studies are warranted to investigate the long-term effects of structured nutrition therapy in patients with type 2 diabetes.
